# Electroencephalogram signals emotion recognition based on convolutional neural network-recurrent neural network framework with channel-temporal attention mechanism for older adults

**DOI:** 10.3389/fnagi.2022.945024

**Published:** 2022-09-21

**Authors:** Lei Jiang, Panote Siriaraya, Dongeun Choi, Fangmeng Zeng, Noriaki Kuwahara

**Affiliations:** ^1^Graduate School of Science and Technology, Kyoto Institute of Technology, Kyoto, Japan; ^2^Faculty of Informatics, The University of Fukuchiyama, Kyoto, Japan; ^3^College of Textile Science and Engineering, Zhejiang Sci-Tech University, Hangzhou, China

**Keywords:** electroencephalogram (EEG), emotion recognition, channel-temporal attention, CNN-RNN, older adults

## Abstract

Reminiscence and conversation between older adults and younger volunteers using past photographs are very effective in improving the emotional state of older adults and alleviating depression. However, we need to evaluate the emotional state of the older adult while conversing on the past photographs. While electroencephalogram (EEG) has a significantly stronger association with emotion than other physiological signals, the challenge is to eliminate muscle artifacts in the EEG during speech as well as to reduce the number of dry electrodes to improve user comfort while maintaining high emotion recognition accuracy. Therefore, we proposed the CTA-CNN-Bi-LSTM emotion recognition framework. EEG signals of eight channels (P3, P4, F3, F4, F7, F8, T7, and T8) were first implemented in the MEMD-CCA method on three brain regions separately (Frontal, Temporal, Parietal) to remove the muscle artifacts then were fed into the Channel-Temporal attention module to get the weights of channels and temporal points most relevant to the positive, negative and neutral emotions to recode the EEG data. A Convolutional Neural Networks (CNNs) module then extracted the spatial information in the new EEG data to obtain the spatial feature maps which were then sequentially inputted into a Bi-LSTM module to learn the bi-directional temporal information for emotion recognition. Finally, we designed four group experiments to demonstrate that the proposed CTA-CNN-Bi-LSTM framework outperforms the previous works. And the highest average recognition accuracy of the positive, negative, and neutral emotions achieved 98.75%.

## Introduction

### Background

**FIGURE 1:**
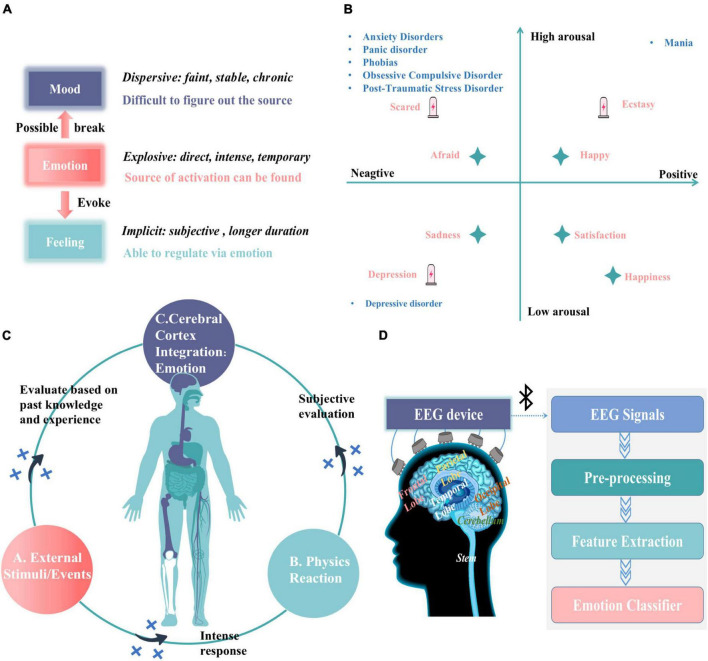
Overview of the causes and methods of monitoring individual (subject) emotions. **(A)** Characteristics of mood, emotion, and feeling. **(B)** Expression of emotions space model and mental disorders. **(C)** The formation of emotion. **(D)** EEG emotion recognition system.

Japanese family norms based on the traditional culture of filial piety form a social support network centered on kinship ties, which differs sharply from the individual-centric social networks of Western countries (Sugisawa et al., 2002; Knight and Sayegh, 2010). As a result, Japanese older adults are more likely to feel socially isolated at a rate of 15.3% compared to 5.3% in the UK (Noguchi et al., 2021). Poor interaction and lack of social participation are among the contributing factors to social isolation which are closely associated with depression, one of the major risk factors for the development of Alzheimer’s dementia (Santini et al., 2015). Many studies (Westermann et al., 1996; Thierry and Roberts, 2007; Sitaram et al., 2011; Iwamoto et al., 2015; Leahy et al., 2018) have shown that reminiscence and communication about past photographs between older adults and younger volunteers, healthcare workers, or families encourage positive interaction and social engagement. And therefore, they are highly effective in improving the emotional state and alleviating depression in older adults. However, it is necessary to evaluate the emotional state of the older person when talking about the photographs to (a) ensure that the communication is positive, as long-term negative emotions may cause changes in feelings and state of mind (Figure 1A) leading to various mental illnesses (Van Dis et al., 2020). For example, mania is easily caused by a prolonged state of ecstasy (high positive) and euphoria (high arousal) as shown in Figure 1B and (b) estimate whether the photographs in the conversation are effective in improving the emotion of the older person, and replace them with other photographs if they are not effective. While numerous studies focus on the evaluation of emotions in older adults, earlier studies generally used self-assessment in the form of verbal or questionnaires and were found to be intermittent and influenced by social expectations or demand characteristics (the idea that participants or stimulators will develop similar or specific emotions in response to perceived expectations) (Orne, 1962). Later developments use smart wearable devices (physiological signals) (Kouris et al., 2020), facial expression (Caroppo et al., 2020), and speech recognition (Boateng and Kowatsch, 2020) to monitor and recognize emotions. However, variances and continuities such as facial aging in older adults and differing accents among various groups of people (e.g., different dialects spoken throughout Japan) make it difficult to distinguish and unify such features and expressions. These inevitably result in unreliable emotion recognition results for older adults.

Thus, while physiological signals to monitor emotions seem to be a more suitable approach for older adults, not all physiological signals are suitable for distinguishing between different emotional experiences. For example, although excitement and panic are different emotions generated in response to different stimuli of award and threat, both exhibit the same physiological changes (i.e., increased heart rate, increased blood pressure, body shaking, etc.). Moreover, time is also necessary for the autonomic and sympathetic nervous systems to switch on and off, resulting in outward physiological changes that are slow-acting and insufficient in keeping up with the emotional changes (Liu and Cai, 2010). By conducting the EEG signals through the electrodes on the scalp we can collect EEG signals with a high temporal resolution that reflect different emotional states and variances between these moments (Alarcao and Fonseca, 2017) (Note: all commercially available acquisition devices have a sampling rate of at least 160 Hz/s). As we know from the widely accepted cognitive-evaluation theory of emotions (two-factor theory) (Cornelius, 1991), when we are stimulated by the external environment, we immediately generate physical reactions and simultaneously evaluate them with past knowledge and experience (cognitive process) and finally integrate them into the cerebral cortex obtaining the emotional state (the whole process shown in Figure 1C). Therefore, we can say that EEG signals have a significantly stronger association with emotions than other physiological signals. They are also objective, non-invasive, and safe.

The general process and principles of EEG signals for the emotion recognition system (shown in Figure 1D) are (1) stimulus materials elicit emotions in the subject while collecting EEG signals, (2) the computer sequentially preprocesses and extracts features from the received EEG signals, and (3) an EEG-based emotion recognition classifier is trained using task-relevant EEG features. The emotion label of EEG features in training emotion classifiers is based primarily on the SAM scale using the valance-arousal emotion model proposed by Posner et al. (2005). The subject is exposed to stimuli and their emotional state is evaluated by oneself using the SAM scale (Valence: positive to negative emotional state; Arousal: difference in the level of physiological activity and mental alertness), which is mapped to the valence-arousal emotion model (Figure 1B) to obtain a corresponding “emotion label.” In this way, the subjective experience of different emotions (emotion labels) and subjects’ objective physiological responses (EEG signals) are matched one-to-one. Nowadays, many inexpensive solutions for portable EEG acquisition devices are available on the market (Stytsenko et al., 2011; Surangsrirat and Intarapanich, 2015; Athavipach et al., 2019), and thus EEG signal-based emotion recognition has a promising application and research value. For this study in the conversation scenario using EEG signals for emotion recognition is extremely challenging. Especially, as the facial muscle activity during the conversation will evoke high-energy artifacts that may distort the intrinsic EEG signal. Such artifacts will hide the rhythm of the real EEG signal and cause perturbation in an EEG system that makes EEG signal processing difficult in all respects (Kamel and Malik, 2014). Therefore, in EEG-based emotion recognition, appropriate signal pre-processing methods must be first adopted to remove artifacts and make the EEG data as clean as possible simply reflects the brain’s activity. Meanwhile, the challenge to reduce the number of dry electrodes to improve user comfort while ensuring a high emotion recognition rate remains.

In this paper, we propose a CNN-RNN framework combined with a channel-temporal attention mechanism (CTA-CNN-Bi-LSTM) for EEG emotion recognition inspired by the channel-spatial attention module (CBAM) proposed in the field of computer vision research (Woo et al., 2018). The primary contributions of this study are summarized as follows.

(1) In the EEG signal pre-processing stage, due to the EMG and EOG artifacts contribute differently to different brain regions and attenuate as the distance from the scalp gets more remote. We divided the 8-channel EEG signals into Frontal, Temporal, and Parietal groups according to brain regions. And then remove multiple biological artifacts from raw EEG signals in each group separately based on the MEMD-CCA method (Xu et al., 2017; Chen et al., 2018).

(2) In the phase of assigning emotional labels to EEG signals, the emotion labels (positive, neutral, and negative) of EEG signals were automatically obtained by the K-means method based on the ratings of the emotion scale [Valence (-4,4), Arousal(-4,4) and Stress (1,7)] of each participant. The advantage of using this method is not to use the same rating classification criteria for all participants, but to use each participant’s rating to classify their own emotions.

(3) For data-driven EEG-based emotion recognition without feature engineering, we developed a CTA-CNN-Bi-LSTM framework. This framework integrates the channel-temporal attention mechanism (CTA) into the CNN-Bi-LSTM module to explore using spatial-temporal information of different important channels (channel attention) and time points (temporal attention) of EEG signals to achieve EEG-based emotion recognition. And the proposed framework achieved average emotion recognition accuracy of 98%, 98%, and 99% in the negative, neutral, and positive emotions.

(4) We conducted four group experiments on the OCER dataset to explore the contribution of each module to EEG-based emotion recognition. The experimental results indicate that the CNN module provided the largest contribution to the accuracy improvement (21.29%) of the proposed framework, the Bi-LSTM module after the CNN module provided little enhancement (8%) of the framework and the addition of the Channel-Temporal attention module before the CNN-RNN module led to a further significant improvement (11%).

### Related works

In this part, we first describe the artifacts that typically emerge during EEG acquisition and existing effective methods to remove them. Then we introduce EEG emotion recognition systems which have evolved from traditional hand-crafted feature extraction to end-to-end deep learning frameworks with channel selection mechanisms.

#### Electroencephalogram artifacts and removal methods

**FIGURE 2:**
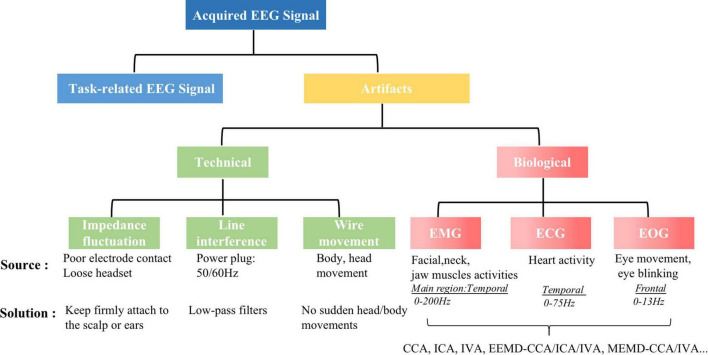
The sources of artifacts in EEG signal and the removal methods.

Due to the potential technical and biological artifacts (Figure 2) in the EEG acquisition process will cause the oscillating discharge larger than the neuronal discharge (Kamel and Malik, 2014). Before proceeding with electroencephalography (EEG) data analysis, it is important to make sure that the EEG data is as clean as possible, meaning that the data collected simply reflects the brain’s activity.

Technical artifacts mainly include three types: impedance fluctuation (Rodriguez-Bermudez and Garcia-Laencina, 2015), line interference (Huhta and Webster, 1973), and wire movement (Urigüen and Garcia-Zapirain, 2015). Technical artifacts can be avoided by paying attention during the acquisition of the EEG signals. Biological artifacts mainly include two types: muscular artifacts [Electromyogram (EMG), Electrocardiogram (ECG)], ocular artifacts [Electrooculogram (EOG)] including eye movement and eye blinking. Such biological artifacts are inevitable contaminations due to the conductivity of the scalp (Kamel and Malik, 2014), and the closer the artifact’s sources are to the electrodes, the more significant is their effect on the EEG data. In particular, the activity of the facial muscles (forehead, cheeks, mouth), neck muscles and jaw musculature (EMG) have a serious effect on the EEG, with a broadband frequency distribution of 0–200 Hz (Halliday et al., 1998; Van Boxtel, 2001). In addition, the heart also is muscular (ECG) and continuously active, which also affects the quality of the EEG data. The artifact has a broadband frequency distribution of 0–75 Hz (Lee and Lee, 2013), but has less effect on the EEG because of the large distance between the scalp and the heart. The eyes have a powerful electromagnetic field, which is formed by millions of neurons in the retina, thus eye movement (horizontal, vertical, and rotation) and eyeblink will affect the electric field received by the electrodes resulting in electrooculogram (EOG) artifacts. Similar to eye movements, eye blinking can interfere with brain signals to a large extent, one, due to the proximity of the eye to the brain, two, as individuals would blink 20 times per minute to keep the ocular moisture of their eyes (Karson, 1983), and these artifacts are unavoidable for prolonged tasks.

Therefore, for our task, the removal of EMG and EOG artifacts from raw EEG signals can be considered the top issue to address. There are already many algorithms (Narasimhan and Dutt, 1996; Jung et al., 2000; Schlögl et al., 2007; Ferdousy et al., 2010; Vos et al., 2010; Safieddine et al., 2012; Sweeney et al., 2012; Teng et al., 2014; Zhao et al., 2014; Chen et al., 2017; Paradeshi et al., 2017; Yang et al., 2017) for removing these two artifacts (summarized in Table 1), the BSS-based techniques are widely proposed because they do not require a priori knowledge and reference electrodes for EMG/EOG signals acquisition and they could separate related artifacts from EEG signal by statistical inference. Among them, CCA-based methods which more effective than ICA-based methods and other filters, taking advantage of the fact that the autocorrelation coefficient of EEG is larger than that of EMG, so it is possible to separate task-related EEG and EMG artifacts. Moreover, relevant studies (Vos et al., 2010; Urigüen and Garcia-Zapirain, 2015) have demonstrated the effectiveness of the CCA method in removing muscle artifacts during speech. EEMD-CCA (Sweeney et al., 2012) is one of the best methods the for removal of EMG and EOG artifacts for single-signals EEG signals. Although for non-single channel EEG signals, EEMD-CCA can be applied channel-by-channel, the inter-channel correlation is not captured. The later proposed MEMD-CCA (Chen et al., 2017) addressed the challenge by decomposing all channels together and then aligning the same frequency components of each channel to form multivariate IMFs before applying CCA (by setting the autocorrelation coefficient threshold, generally less than 0.9 components are set to 0) to remove the artifacts to reconstruct the EEG signals. However, it does not take into account the different degrees of influence on the EEG signals due to the distance of the artifact source from the location of the scalp electrodes (shown in Figure 2). Therefore, it is necessary to group the EEG channels based on brain areas and then use MEMD-CCA on each group separately.

**TABLE 1:** Comparison of EMG and EOG artifacts removal techniques.

Methods		Ref. E	Channel	Comparison results
PK	NPK (BSS-based)			(Better than)
Adaptive filtering		√	All	EMG: Low-pass filter EOG: WPT, ICA, DWT, ANC (Narasimhan and Dutt, 1996; Zhao et al., 2014)
Linear regression		√	All	EOG: Visual identification (Schlögl et al., 2007)
	ICA	×	Multi	PCA, LR, Wavelet (Jung et al., 2000; Paradeshi et al., 2017)
	CCA	×	Multi	EMG: low-pass filter + Robust ICA; EOG: equivalent to ICA (Ferdousy et al., 2010; Vos et al., 2010)
	EMD	×	Single	ICA, CCA, WT (Safieddine et al., 2012)
	EEMD-CCA	×	Single	EMD, EMD-ICA, EMD-CCA, EEMD, EEMD-ICA (Sweeney et al., 2012)
	MEMD	×	Few	ICA (Teng et al., 2014)
	MEMD-CCA	×	Few	EMG: ICA, EEMD-ICA, MEMD-ICA CCA, EEMD-CCA (Chen et al., 2017)
	CCA-MEMD	×	Few	EOG:ICA, CCA (Yang et al., 2017)

PK, prior knowledge; NPK, no prior knowledge; BSS, blind source separation; Ref. E, reference electrode; ICA, independent component analysis; CCA, canonical correlation analysis; EMD, empirical mode decomposition; EEMD, ensemble empirical mode decomposition; MEMD, multivariate empirical mode decomposition.

#### Electroencephalogram emotion recognition systems

Electroencephalogram emotion recognition systems, mainly differ in their approach to feature extraction and choice of classifiers: a step-by-step machine learning framework (hand-crafted feature extraction, feature fusion, modeling classification) and an end-to-end deep learning framework (automatic feature extraction, feature fusion, modeling classification).

##### Step-by-step machine learning framework

The performance of machine learning frameworks largely depends on the quality of hand-crafted extracted features (Hosseini et al., 2020). Generally, researchers extract the EEG features from parts of the brain regions considered to contribute the most to emotions based on *a priori* knowledge of the combinatorial design. Of the most used in emotion recognition are the following two theories based on asymmetric behavior: (1) the right hemisphere dominance theory which posits right hemispheric dominance over the expression and perception, and (2) the valence theory which asserts that the right hemisphere predominantly processes negative emotions and left hemisphere predominantly processes positive emotions (Coan and Allen, 2003; Demaree et al., 2005). For example, in the study (Wang et al., 2014), the authors subtracted the power spectrum (PSD) of obtained brain waves collected from 27 pairs of symmetrical electrodes in the left and right brain regions to obtain 27 asymmetrical PSD features input to SVM classifiers. The negative and positive emotion recognition accuracy average rate was 82.38%. Later studies (Duan et al., 2013; Zheng et al., 2014, 2017) demonstrated that the following six features: PSD, differential entropy (DE), DASM (DE(L_eft_)-DE(R_ight_)), RASM (DE(L_eft_)/DE(R_ight_)), ASM ([DASM, RASM]), DCAU(DE(F_rontal_)-DE(P_osterior_)) were robust and effective for EEG emotion recognition. However, the DE features achieved the highest recognition accuracy of 91.07% which is higher than the other four asymmetric features. This demonstrates ambiguity as to what degree the stimuli (pictures, music, videos, etc.) elicit neuronal processes similar to those occurring in real-life emotional experiences; making it difficult to cover all the implied features by hand-extracted features.

##### End-to-end deep learning framework

Recently studies began to focus on end-to-end deep learning frameworks (Craik et al., 2019). In a study (Alhagry et al., 2017), the authors proposed the use of LSTM models to automatically learn features of emotions from the context of EEG signals. They achieved average recognition accuracy of 85.65% in the valence dimension. Later in a study (Zhang et al., 2020) the authors further considered that the spatial information in the EEG signal could be used to improve the accuracy of emotion recognition and thus proposed a CNN-LSTM model. EEG raw data was first input into a CNN module (1-dimensional convolutional layer, maximum pooling layer) to extract the local spatially features which were then input into a two-layer LSTM to learn the temporal information in the spatial features. The result was an average recognition accuracy of 94.17% with a four-emotion classification. In addition, the authors input EEG raw data separately into CNN (four-layer of two-dimensional convolution; spatial features) and LSTM (four-layer; temporal features) to achieve accuracies of 90.12% and 67.47%, respectively. A later study (Sheykhivand et al., 2020) also proposed the use of CNN-LSTM for EEG raw data with the main structure of 10-1D convolutional layers plus 3 LSTM layers, achieving a recognition average accuracy of 97.42% with a two emotion classification.

From the results of the above-related studies, it was found that (a) the model automatically learns emotional features from EEG raw data better than hand-crafted extracted features, and (b) the model emotion classification recognition performance using EEG spatial-temporal features demonstrates improvements across a wide range. In addition, there are also studies that combine feature extraction and deep learning models, such as a DECNN model (Liu et al., 2020) was proposed that focuses on subject-independent emotion recognition and used extracted DDE (dynamic differential entropy) features fed into the CNNs for emotion classification. Finally, the average accuracy achieved 97.56% in EEG subject-independent emotion recognition on the SEED public dataset.

#### Channel selection mechanism

The number of dry electrodes used in the EEG emotion recognition systems studied above is, in general, excessive and not conducive to prolonged wear from a comfort perspective. Moreover, the EEG signals obtained with multichannel EEG devices often contain redundant, irrelevant, or interfering information (noise, overlapping/interference of signals from different electrodes) for affective analysis (Alotaiby et al., 2015). Thus, selecting the most relevant channel for emotion analysis is essential for enhancing comfort and emotion recognition accuracy.

A study (Tong et al., 2018), utilized the Relief algorithm to calculate the weight values of each channel according to the time-domain features of the EEG signal. At the cost of losing 1.6% accuracy, 13 channels with the highest contribution to emotion classification under time-domain features were selected from the initial 32 channels. Later, a study (Dura et al., 2021) used the reverse correlation algorithm applied to the band-time-domain features of 32 channels to construct a subset of electrodes with the smallest band correlation for each subject. The number of occurrences of each subset in each subject was then calculated to obtain the most common subset of channels. The smallest subset contained only four electrodes and accuracy was not affected. However, the accuracy of such channel selection methods would depend entirely on the quality of hand-extracted features. In response, the latest has research proposed to apply an attention mechanism to channel selection to prompt the network to automatically learn the most important information and improve the performance of important features. In a study (Tao et al., 2020), the authors added the channel attention module before the CNN-LSTM model to automatically learn the importance of each channel to the EEG emotion signals and then assigned weights to each channel. It was found that the FC5, P3, C4, and P8 channels contributed the most to emotion classification on the DEAP dataset (32 channels) and had an average accuracy improvement of 28.57% compared to the CNN-LSTM model without the channel attention. Later, a 3DCANN (Liu et al., 2021) framework was proposed, in which five consecutive 1s-62-channel EEG signals were fed as 3D data inputted to a CNNs module with two convolutional layers to extract spatial features, which were later output to two attention modules in the channel dimension to enhance or weaken the effect of different electrodes on emotion recognition. The model achieved an average accuracy of 96.37% for positive, negative, and neutral emotions. It is demonstrated that the attention mechanism enhances the information of the important channels and suppresses the information of the irrelevant channels for emotion analysis. However, the shortcoming is that, to get the global perspective of the temporal dimension (Time × Sample point) of the EEG signals (Time × Sample point × Channel), the channel attention module pools the EEG signals globally into 1 × 1 × Channel to get the weight matrix of the channel. This directly ignores the specific temporal information of the EEG signals, if a channel contains more noise/artifacts, it may get larger weight values instead of being conducive to the later model learning.

As our purpose is to perform emotion recognition during the conversation, even if the removal of artifacts is implemented in EEG signals, some artifacts may be still present. Therefore, attention mechanisms need to be applied simultaneously in the temporal dimension. The temporal attention mechanism will play an important role in determining “where” the need to focus attention exists. It can improve the expressiveness of the time points of changing emotional states in the EEG signal while suppressing noise/artifacts information.

## Materials and methods

In this section, first, we describe the EEG dataset, the method of division of the EEG dataset, and the preprocessing of EEG signals. Then, we describe in detail the structure of each module of the proposed CTA-CNN-Bi-LSTM.

### The division and preprocessing of electroencephalogram dataset

Our experiments were conducted on the dataset from the previous study (Jiang et al., 2022). Eleven older adults (six males and five females) and seven younger adults (five males and two females) were randomly pair-matched into 11 groups, and each group engaged in 36 photo conversations. The young person guided the older adult in a 1-min conversation around each photo during which time the EEG signals from the older adult were collected. After each photo conversation, the older adult also filled out an emotion evaluation form (rating of valence, arousal from –4 to 4, and the level of stress from 1 to 7). A detailed description of the EEG dataset (here named OCER) is presented in Table 2.

**TABLE 2:** Summary of experiment dataset (OCER).

Conversation experiment	
Trails	36 trails × 60 s
Subject	Older: 11 (*M* = 71.25 ± 4.66) Young: 7 (*M* = 22.4 ± 1.51)
Rating	Valence (–4,4), Arousal (–4,4), Stress (1,7)
**EEG dataset**	
Device	OpenBCI Cyton board (250 Hz/s)
Channel	F3, F4, F7, F8, T7, T8, P3, P4 (10–20 system)
Array	396(Samples) × 60 (s) × 250(Hz/s) × 8 (Channels)

As individual differences in gender, age, economic, educational, and life circumstances would result in differences in the benchmarks for evaluating emotions, we did not classify samples by uniformly setting thresholds for the rating values on each dimension. Instead, in our experiments, the ratings of valence, arousal, and stress were first standardized using the standard deviation standardization method (*Z*-score). And the K-means method (Likas et al., 2003) was then applied to the three standardized scores for each individual, and the 36 samples were divided into three groups of positive, neutral, and negative emotions samples. Finally, the pre-processing was applied to the EEG signals in the dataset:

#### Removal of technical artifacts

Electroencephalogram signals used a 1–45 Hz bandpass filter (removal of the line interference) and a Chebyshev I high-pass filter to remove baseline drift (Jiang et al., 2022).

#### Removal of biological artifacts

**FIGURE 3:**
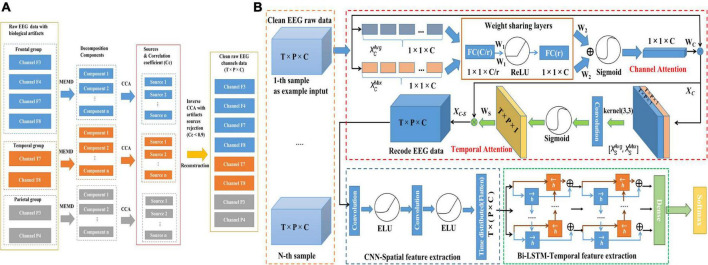
Illustration of the proposed framework based on raw EEG data for emotion recognition. **(A)** Removal of biological artifacts by MEMD-CCA. **(B)** CTA-CNN-Bi-LSTM framework.

Electroencephalogram signals were divided into three groups: The frontal group (F7, F8, F3, F4), the temporal group (T7, T8), and the parietal group (P3, P4). And then each group used MEMD-CCA (Xu et al., 2017; Chen et al., 2018) methods to remove multiple artifacts (detailed in Figure 3A).

#### Segmented data

Used a 3s-non-repetitive window for segmentation of each sample (60 s trail) in the dataset. The reason is that normally the duration of an adult’s emotional state does not exceed 12 s and in studies (Li et al., 2017; Tao et al., 2020) a 3s-non-repetitive sliding window applied to the EEG signals achieved excellent results for the emotion recognition task.

### Preprocessing of proposed framework

First of all, before inputting the raw clean EEG dataset into the first module of the proposed framework, we normalized each raw EEG sample along the channel direction with zero-mean normalization to eliminate subject and channel differences in EEG signals and reduce computational complexity. Thus, the mean value of the processed raw EEG signals sample for each channel is 0 and the standard deviation is 1. The normalization formula for each channel is as follows:


(1) $$X_{i,j}^{k*}=\frac{X_{i,j}^{k}-\bar{X^{k}}}{\sigma^{C}}.$$

Where $X_{i,j}^{k}\left(i=1,2,3Time\left(second\right);j=1,2,\ldots ,250\;SamplePoint\left(250Hz/s\right);k=1,2,\ldots ,8EEGChannel\right)\in R^{T\times P}$ represents the data of the k-*th* channel of a 3s-EEG sample. T and P are the time length and the sample point of the 3s-EEG sample respectively. $\bar{X^{k}}$ and σ*^C^* are the mean and standard deviation of the k-th channel respectively.

### Modules of proposed CTA-CNN-Bi-LSTM framework

The proposed framework consists of the following three modules: channel-temporal attention module, spatial feature extraction module (CNNs) and bi-directional temporal feature extraction module (Bi-LSTM). The structure of the proposed CTA-CNN-Bi-LSTM framework is shown in Figure 3B. The specific calculation process and description are as follows.

#### Channel-temporal attention module

An EEG sample is defined as *X* ∈ *R*^*T*×*P*×*C*^ whereby *T* denotes the time duration of one EEG sample, *P* is the sampling points per second and C denotes the number of EEG channels. The output after the channel-temporal attention module is *X*_*c* −*s*_ ∈ *R*^*T*×*P*×*C*^, and the specific calculation process and descriptions are as follows. Here for our EEG dataset, the *T* is 3 s, *P* is 250 Hz/s, and *C* is 8 channels.

##### Channel attention

The global average pooling and maximum pooling are performed separately in the temporal dimension on the channel direction of *X* to obtain two channel statistical descriptions $X_{C}^{\text{Avg}},X_{C}^{\text{Max}}\in R^{1\times 1\times C}$. They are then fed into a two-layer weight-sharing multi-layer perceptron (MLP): the first layer is the compression layer (the number of neurons is set to C/r to get the weight *W*_1_ ∈ *R*^1×1×*C*/*r*^ and ReLU is used as the activation function. *r* represents the reduction ratio and here *r* is set to 2); The second layer is the excitation layer (the number of neurons is set to C to get the weight *W*_2_ ∈ *R*^1×1×*C*^). Finally, these two combined features are mapped using a sigmoid activation function to generate the channel attention mapping matrix *W_c_* ∈ *R*^1×1×*C*^ as follows:


(2) $$\begin{array}{c} W_{c}\left(\text{X}\right)=\text{Sigmoid}(W_{2}(\text{ReLu}\left(W_{1}\cdot X_{c}^{\mathrm{Avg}}\right)+\\ \;W_{2}\left(\text{ReLu}\left(W_{1}\cdot X_{c}^{\mathrm{Max}}\right)\right)). \end{array}$$

And the output of channel attention *X*_*c*_ ∈ *R*^*T*×*P*×*C*^ is as follows:


(3) $$X_{c}=W_{c}\left(X\right)⊗X.$$

##### Temporal attention

Average pooling and maximum pooling are used along the channel dimension on the temporal direction to obtain $X_{\text{s}}^{\text{Avg}},X_{\text{s}}^{\text{Max}}\in T\times P\times 1$ to stitch them together, and convolutional layers (a convolutional kernel of size 3 × 3, *K*^3×3^). The sigmoid activation functions are used to generate the temporal (*T*×*P*, Time × Sample Point) attention mapping matrix *W*_*s*_ ∈ *R*^*T*×*P*×1^ as follows:


(4) $$W_{s}\left(X\right)=Sigmoid\left(K^{3\times 3}\left(\left[X_{s}^{\mathrm{Avg}},X_{s}^{\mathrm{Max}}\right]\right)\right).$$

Thus, our final output *X*_*c*−*s*_ ∈ *R*^*T*×*P*×*C*^ is as follows:


(5) $$X_{c-s}=W_{s}\left({\text{X}}_{\text{c}}\right)⊗X_{c}.$$

In this way, the output shape of *X*_*c*−*s*_ ∈ *R*^*T*×*P*×*C*^ remain unchanged and has learned what channels are important and at which time points in the channel and temporal dimension.

#### Convolution neural networks module

The convolution neural networks (CNNs) and their essential characteristics (spatially dependencies/local connection and weight sharing) have been widely used in various fields, especially for image tasks (object segmentation, image classification, style conversion, etc.) (Hijazi et al., 2015; Rawat and Wang, 2017). All of these applications were built based on the feature maps after the CNNs performed feature extraction for the task. Thus, essentially the role of CNNs models is to extract local spatial features of EEG signals. The specific steps of our CNNs module are as follows.

##### Step 1: Convolution layer

The input recorded EEG signals *X*_*c*−*s*_ ∈ *R*^*T*×*P*×*C*^ and the convolution kernel of CNNs is defined as ${\mathrm{filter}}_{\left(i,j\right)}^{k}$. *k* represents the number of filters, which is the same as the number of EEG channels. (*i*, *j*) is the size of the convolutional sliding window in the temporal-spatial (*T*×*P*, Time × Sample Point) dimension of multi-channel EEG signals. More specifically, the *k*-th filter is convolved with the corresponding region in *T*×*P* dimension of the *k*-th channel of *X*_*c*−*s*_ with a window size of *i*×*j* sliding in step 1 (direction from left to right and top to bottom). The output value is obtained by adding the sum of the *k* channels. Finally, the feature map $X_{C-S}^{f}$ is as follows:


(6) $$X_{C-S}^{f}=f\left(\sum X_{C-S}⊗K+b\right).$$

The bias term is represented by *b*. A convolution kernel produces a feature map, and the closer the value in $X_{C-S}^{f}$ is to 1, the more it is associated with the feature, and the closer it is to –1, the less it is associated.

In our dataset for EEG emotion recognition, the k was set 8 corresponding to the number of EEG channels. And the size of the sliding window (*i*, *j*) was set “1×10” and sliding in step 1, where the “*i*” was set 1 in order not to destroy the temporal features of the EEG signals at different seconds and the convolutional window to constantly move at the same second as the sampling points when sliding. Therefore, later the generated spatial feature maps have the following characteristics: (a) different spatial locations on the same channel were sharing convolutional kernel parameters (spatial independence), and (b) different convolutional kernels were used on different channels (channel specificity). This allowed each feature map of the output CNNs to learn different spatial emotion features with temporal information preserved.

##### Step 2: Exponential linear units layer

The exponential linear units (ELU) was selected as the activation function after the convolution layer because it is continuous and differentiable at all points and its gradient is non-zero for all negative values, meaning it does not encounter the problem of exploding or disappearing gradients during deep network learning. It achieves higher accuracy compared with other activation functions such as ReLU, Sigmoid, and tanh (Clevert et al., 2015). The ELU activation function can be written as:


(7) $$f\left(x\right)=\left\{ \begin{array}{c} e^{x}-1,x<0\\ x,x\geq 0 \end{array}\right..$$

As can be deferred from (7), the ELU function retains the values greater than or equal to 0 in the feature map $X_{C-S}^{f}$ and assigns *e^x^*−1 to all the remaining values less than 0. This further suppresses the uncorrelated data in the feature map using a non-linear activation function.

To ensure that the temporal information contained in the extracted spatial feature maps is not reduced during the input temporal feature extraction module (Bi-LSTM), we did not use the pooling layer often used in CNN structures. Thus, our spatial feature extraction module used two convolution-ELU layers.

#### Bi-directional long short-term memory module

LSTM networks (Hochreiter and Schmidhuber, 1997) have been widely used in time series related tasks, such as disease prediction (Chimmula and Zhang, 2020) and air quality prediction (Yan et al., 2021). This is because, unlike previous feedforward neural networks (one-way propagation, where the input and output are independent of each other), LSTM networks have internally inclusive memory units (the state of the current time step is jointly determined by the input of that time step and the output of the previous time step). LSTM is, effectively, a gating algorithm added to the memory unit of a traditional RNN which solves the problem of long sequences in which the gradient disappears and explodes during the training process of the RNN model (Bengio et al., 1994). The memory unit of LSTM is as follows:


(8) $$Z_{forget}=sigmoid\left(W_{f}\left[h_{t-1},X_{t}\right]+b_{f}\right),$$

(9) $$Z_{input}=sigmoid\left(W_{i}\left[h_{t-1},X_{t}\right]+b_{i}\right),$$

(10) $$Z_{output}=sigmoid\left(W_{o}\left[h_{t-1},X_{t}\right]+b_{o}\right),$$

(11) $$Z=tanh\left(W\left[h_{t-1},X_{t}\right]+b_{c}\right),$$

(12) $$C_{t}=Z_{forget}C_{t-1}+Z_{input}Z,$$

(13) $$h_{t}=Z_{output}tanh\left(C_{t}\right),$$

(14) $$y_{t}=\sigma \left(W_{t}h_{t}\right).$$

where *Z*_*forget*_, *Z*_*input*_, *Z*_*output*_ are vectors of data input from the current state and input data received from the previous node multiplied by the weights and then converted to values from 0 to 1 by a sigmoid activation function which acts as a gating function (0 means complete discard of information, 1 means complete retention of information). Thus *Z*_*forget*_ determines which information in *C*_*t*–1_ needs to be forgotten; *Z*_*input*_ determines which new information in *X*_*t*_ needs to be recorded; *Z*_*output*_ determines which information is the output of the current state. Z is converted to a value between –1 and 1 by the tanh activation function as the input data for *C*_*t*_. In addition, *C*_*t*_ (cell state) and *h*_*t*_ (hidden state) represent the two transmission states of the memory cell to the next cell of the LSTM. *y*_*t*_ is obtained from *h*_*t*_ by σ transformation and represents the output of the memory cell.

However, for the EEG emotion recognition task, the current emotional state is correlated with both previous and subsequent information due to the latency of the device during signal acquisition. Bi-LSTM (Schuster and Paliwal, 1997) is an extension of LSTM consisting of a forward LSTM layer (fed the sequence, left to right) and a backward LSTM layer (reversed fed the sequence, from right to left) which can solve this problem. The out layer of the memory unit of Bi-LSTM is as follows:


(15) $$y_{t}=\sigma (W_{t}\left(\underset{h_{t}}{\to }+\underset{h_{t}}{\leftarrow }\right).$$

In addition, the LSTM contains overly numerous parameters, and the Bi-LSTM is twice as large as the LSTM, so it is easy to overlearn to produce the overfitting problem. The most common solution in deep learning is the utilization of dropout regularization (Hinton et al., 2012) which temporarily disconnects the input-hidden layer-output layer with a certain probability. However, temporarily dropping layer-to-layer connections in recurrent neural networks may cause direct loss of some of the previous memory. Therefore, we use the recurrent dropout method (Semeniuta et al., 2016) to act on the memory units; temporarily dropping a part of the links in *h*_*t*_ (hidden state) with probability p at each time step. This ensures that the output *y*_*t*_ does not lose the earlier important information while simultaneously solving the overfitting problem. Therefore, the features of past and future emotion information through this structure were combined in the out layer. Here, our temporal feature extraction module consists of two layers of internal memory cell units (32 and 16 respectively) with a 0.2 recurrent dropout rate of the bidirectional LSTM.

In summary, our proposed framework can automatically extract meaningful features for emotion classification from raw clean EEG data. Firstly, a channel-temporal attention mechanism is used to infer attention weights for raw EEG signals X successively along the channel and temporal dimensions and got re-coded EEG signals *X*_*c*−*s*_, which improves the points of time representation of significant channel and emotional state changes. Next, CNNs (including two convolution-ELU layers) are used to extract spatial features of *X*_*c*−*s*_ to get feature maps $X_{C-S}^{f}$. Finally, all spatial feature maps $X_{C-S}^{f}$ were packaged in time series input into a two-layer Bi-LSTM with a recurrent dropout function to learn temporal information from the spatial features maps for EEG emotion recognition.

## Results and analysis

Firstly, we describe the division and preprocessing of the EEG dataset. Secondly, we displayed the result of the channel attention weights in the channel-temporal attention module. Finally, we introduce designed four groups of deep learning methods for demonstrating the validity of each module of our proposed method.

### The division and preprocessing of electroencephalogram dataset

The standardization of the scores of the rating scale [Valence (–4,4), Arousal (–4,4) and Stress (1,7)] and the classification of emotions using K-means for 36 samples of each participant was completed in IBM SPSS Statistics (version 26). The related results were displayed in Table 3. Each participant’s 36 trials were divided into three categories respectively: Clustering “1” represented the “negative emotion”; Clustering “2” represented the “neutral emotion”; and Clustering “3” represented the “positive emotion”. The advantage of using this method is that instead of using the equal criteria for all participants, each participant’s criteria was used to classify the emotions. Therefore, there are 73 negative samples, 182 neutral samples and 141 positive samples in the EEG dataset (OCER). Then, after removing the technical and biological artifacts in the EEG dataset by using the method mentioned in section the division and preprocessing of electroencephalogram dataset, we found that artifacts of the 36-th trail from subject 4, the 11-th trail from subject 6, the 31-th and 32-th trails from subject 8, the 23-th trail from subject 9 and the 35-th trail from subject 10 could not be removed cleanly (the amplitude of EEG signals more than 200 μV) and they were excluded. Finally, we cut each clean trail using a 3-s non-repeating window. Therefore, the array of the EEG dataset became 7800 (390 × 20 segments) × 3 (seconds) × 250 (sample points/s) × 8 (channels). The details were shown in Table 4.

**TABLE 3:** Division of OCER into three motions by K-MEANS.

Subject	Rating	Clustering center (*Z*-score)		
ID	scale	1	2	3
1	Valence	–1.58	0.26	1.27
	Arousal	–0.98	0.65	1.85
	Stress	–0.90	–0.90	–0.90
2	Valence	–1.58	0.55	1.16
	Arousal	–0.98	–1.12	1.08
	Stress	–0.90	–0.90	–0.90
3	Valence	0.63	0.61	0.63
	Arousal	–2.40	–0.96	0.44
	Stress	0.23	–0.80	–0.90
4	Valence	–0.20	0.63	1.02
	Arousal	–0.41	–0.56	0.62
	Stress	0.23	0.46	0.38
5	Valence	–1.58	–1.53	–0.11
	Arousal	–3.11	–1.07	–0.98
	Stress	0.23	0.78	1.36
6	Valence	–0.35	0.22	0.63
	Arousal	–0.63	0.44	0.60
	Stress	2.30	0.73	2.01
7	Valence	–3.05	–1.58	–0.48
	Arousal	–0.98	–0.98	–0.98
	Stress	–0.90	–0.83	–0.90
8	Valence	0.14	–0.11	0.35
	Arousal	0.91	–0.27	0.44
	Stress	1.36	–0.90	0.23
9	Valence	–1.39	–0.45	0.51
	Arousal	–0.81	–0.60	–0.20
	Stress	2.78	1.10	0.41
10	Valence	–0.48	0.49	1.36
	Arousal	–0.27	0.91	1.69
	Stress	–0.90	–0.85	–0.90
11	Valence	–0.11	0.63	1.36
	Arousal	0.40	0.76	1.14
	Stress	0.23	0.23	0.23

All results are retained to 2 decimal places. The larger the score of Valence indicates the more positive; the larger the score of Arousal indicates the greater emotional intensity (no positive or negative directionality); the larger the score of Stress indicates the greater stress (negative directionality).

**TABLE 4:** The emotion classification of OCER and the data arrays.

Emotion classification	
Negative	72 samples (60 s)
Neutral	180 samples (60 s)
Positive	138 samples (60 s)
**3s-dataset arrays**	
Dataset	7800(seg) × 3(s) × 250(Hz/s) × 8(channels)
Label	7800 × 3(Negative, Neutral, Positive)

### Electroencephalogram channel attention weights

**FIGURE 4:**
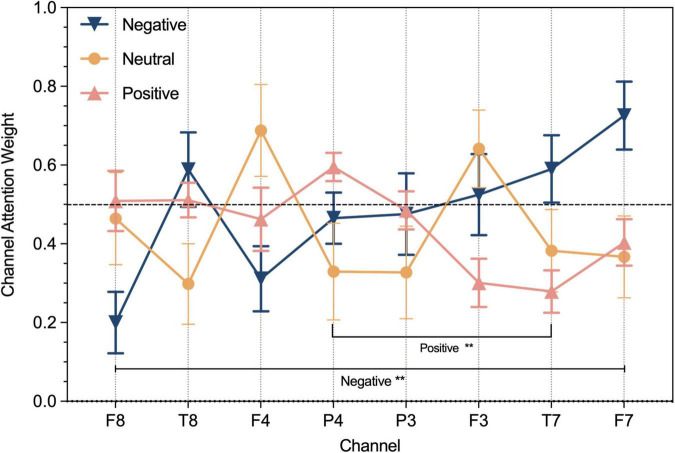
The result of channel weight on OCER dataset respectively for negative, neutral, and positive emotions. **P < 0.01.

To illustrate the different degrees of importance of each EEG signal channel for emotion recognition, the mean of ten times weight calculations of the channel attention in the channel-temporal attention module for OCER are shown in Figure 4. As shown, the weights of the channels for different emotions were significantly different. The EEG signals of the channels corresponding to the right brain regions (except F4 is less than 0.5) contributed more to positive emotions. The EEG signals the channels corresponding to the left brain regions (except P3 less than 0.5) contributed more to negative emotions. And the weights of channel F4 and channel F3 achieved significant advantages in neutral emotion. Further to demonstrate the contribution of different channels to the emotions, the one-way ANOVA was implemented on the 8-channel weights of the three emotions respectively. The weights of channel F8 and F7 had a significant (*F* = *3.55, p* < 0.01) in negative emotion, channel P4 and T7 had a significant (*F* = 3.39, *p* < 0.01) in positive emotion, a non-significant for 8-channel in neutral emotion. This suggests that there are variances in the contribution of channels to different emotions and utilizing channel attention mechanisms could enhance the ability to discriminate between different emotions.

### Parameters of proposed and baseline methods

The proposed framework mainly was implemented with the Keras module based on the TensorFlow framework and trained on NVIDIA GeForce GTX GPU. At first, each batch size (here denoted by None) of samples defined as (None, 3,250,8) was input into the CTA module and the output shape was the same as (None, 3,250,8). The samples were then fed into the CNN module which used the AdaBelief optimizer (Zhuang et al., 2020) with a learning rate of 1e-3 and the epsilon of 1e-7 to minimize the cross-entropy loss function. In order not to destroy the temporal information in the EEG signals, the size of the convolution kernel was set to 1×10×8 (height, width, depth) and the number of kernels was 8 the same as the number of EEG signal channels. This makes the number of channels of the feature maps of the output CNNs consistent with the number of channels of the EEG raw data. This causes each feature map in the input Bi-LSTM with a shape of (None, 3,250,8), which means that the time step is 3 and the features map was split up into three feeds that can be expressed as (None, 3,250 × 8). Therefore, the Bi-LSTM module further extracts the temporal features from the spatial feature maps containing temporal information. In the Bi-LSTM module, the dimension of the hidden states of the LSTM in each direction (forward/backward) of the two layers were 32 and 16, respectively. In addition, in every LSTM the recurrent dropout rate was set as 0.2. Initially, the input batch size is 10 and the epoch is set at 200. And the early stopping technique (Prechelt, 1998) is used during the training process: the training is stopped when the loss value of the test set no longer decreases in two epochs.

To demonstrate the validity of the three modules in the proposed framework, four groups of experiments were implemented: **Group A:** LSTM, Channel Attention-LSTM (CA-LSTM) and Channel-temporal Attention (CTA)-LSTM; **Group B:** Bi-LSTM, CA-Bi-LSTM, and CTA-Bi-LSTM; **Group C:** CNN-LSTM, CA-CNN-LSTM, CTA-CNN-LSTM; **Group D:** CNN-Bi-LSTM, CA-CNN-Bi-LSTM and CTA-CNN-Bi-LSTM. Here the LSTM and Bi-LSTM are referred to generically as RNN. Except for the different number of RNN layers, the model including the CNN-RNN module used two layers of LSTM/Bi-LSTM with hidden states of dimensions 32 and 16. The model including the RNN module used 3 layers of LSTM/Bi-LSTM with hidden states of dimensions 64, 32, and 16. Because each layer of Bi-LSTM is combined with two directions of LSTM, the training parameters are twice as large as the LSTM. All models use the same parameter settings as the proposed method. Specific structures and parameters were shown in Tables 5, 6.

**TABLE 5:** Baseline and proposed method for EEG dataset emotion recognition.

	Channel attention	Temporal attention	CNN	LSTM/Bi-LSTM
RNN	×	×	×	√
C-RNN	√	×	×	√
CTA-RNN	√	√	×	√
CNN-RNN	×	×	√	√
C-CNN-RNN	√	×	√	√
*Proposed method*	√	√	√	√

**TABLE 6:** Array and total parameters of 3s-dataset (OCER) fed into different models.

Model	Input array	Main layers	Total params
RNN	None × 3 × 2000	3 Unit (64,32,16)	544,243/1108,963
C/CTA-RNN	(None × 3 × 250 × 8) reshape (None × 3 × 2000)	3 Unit (64,32,16)	544,243/1108,963
-CNN-RNN	(None × 3 × 250 × 8) reshape (None × 3 × 2000)	2 Conv (*K* = 8(1,10)) 2 Unit (32,16)	648 × 2 + 263411/530915

### Results of experiments

Our work aimed to evaluate individuals’ emotions during the periodic implementation of reminiscence therapy, in which our focus was on the individual’s emotion recognition accuracy, rather than an emotional recognition model to accommodate all older adults. Therefore, the subject-dependent method was utilized for EEG emotion recognition. All samples of the OCER dataset (Table 4) were divided into training sets and test set based on the 10-fold cross-validation method. This method randomly divided the dataset into ten equal parts (nine parts as the training set and one part as the test set) and this process repeated 10 times. Finally, the number of samples in the training set was 7020 and the number of samples in the test set was 780.

**FIGURE 5:**
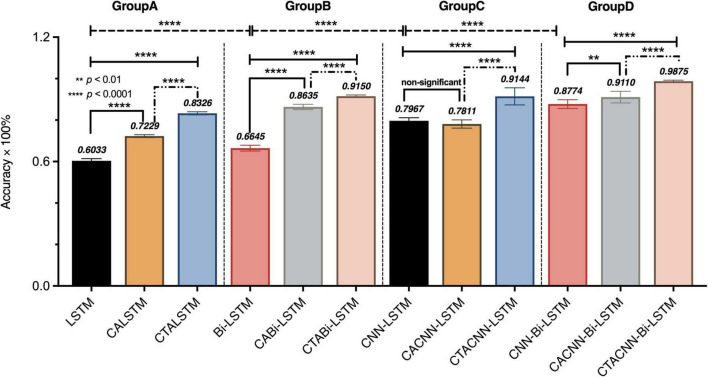
The results of mean accuracy (%) and one-way ANOVA in baseline and proposed methods on 3s-data set. The symbol ** means that *P* < 0.01 and is a statistically significant difference. The symbol **** means that *P* < 0.0001 is an extremely significant statistical difference.

The average accuracy of the results of the 10 test sets was used as the evaluation metric for the performance of the proposed framework and baseline methods. Further, a one-way ANOVA was performed on the results of average emotion recognition accuracy rate for four groups of 12 models to explore whether there was a significant in EEG emotion recognition across models. The detailed results were shown in Figure 5. As seen from the figure, for the average accuracy of emotion recognition on the OCER dataset: (1) Significance among groups A, B, C, D (*F* = 372.8, *p* < 0.0001); (2) Significance among the models within groups A, B, C, D (*p* < 0.01 or *p* < 0.0001), except between the CNN-LSTM model and the CA-CNN-LSTM model in group C (*p* = 0.7756, non-significant). (3) The proposed framework CTA-CNNN-Bi-LSTM in group D achieved the best emotion recognition accuracy with 98.75% on three emotions.

**FIGURE 6:**
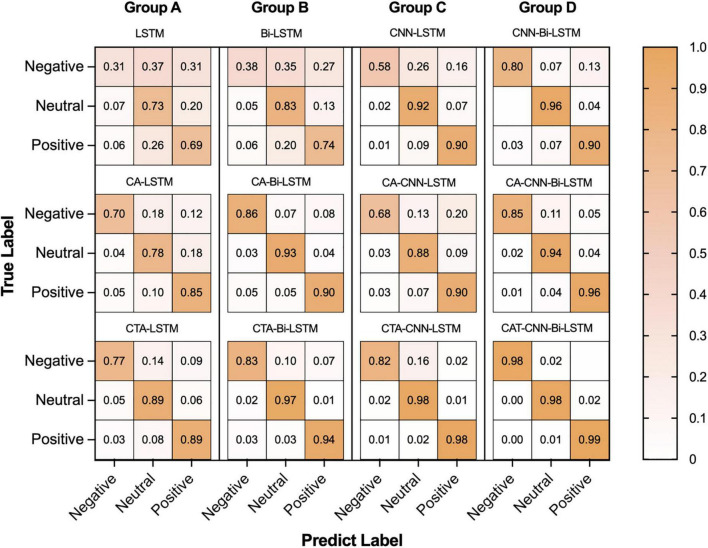
The result of confusion matrixes of negative, neutral, and positive emotions in baseline and proposed method.

To demonstrate the contribution and performance of each module of the proposed framework on the recognition of negative, neutral, and positive emotions, we implemented confusion matrices on all models (see Figure 6). As can be seen, vertically, from the base models (LSTM, Bi-LSTM, CNN-LSTM, CNN-Bi-LSTM) to the front of the models adding the channel attention mechanism (CA) and adding the channel-temporal module (CTA), the recognition accuracy improves for almost in the three emotions. Specifically, the recognition accuracy of negative, neutral and positive emotions improved by at least 18%, 2%, and 9%, respectively. It proved the effectiveness of the CTA module in improving the model’s performance in distinguishing between different emotions. Horizontally, from left to right, from the LSTM series models to the CNN-Bi-LSTM series models (except for the CA-CNN-LSTM model), the recognition accuracy improves for almost the three emotions. Specifically, the recognition accuracy of negative, neutral and positive emotions improved by at least 21%, 9%, and 10%, respectively. It sufficiently demonstrated the superiority of CNN-Bi-LSTM in integrating the bi-directional temporal features (past and future information features) on the spatial features information in the EEG signals information to determine the current emotional state. The CTA-CNN-Bi-LSTM model achieved the best accuracy of emotion recognition for negative, neutral, and positive emotions.

**FIGURE 7:**
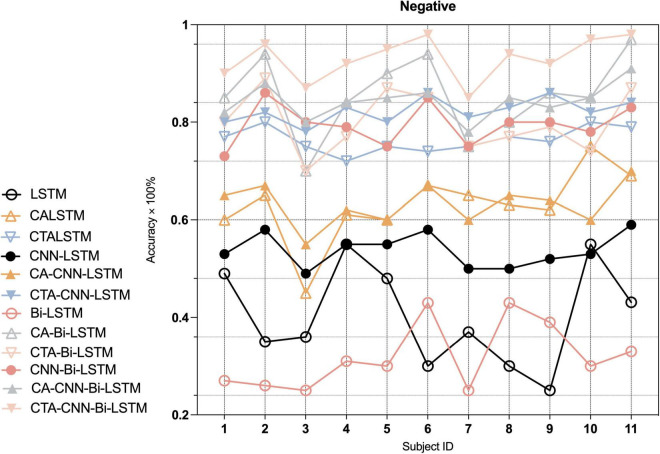
Average accuracy (%) of baseline and proposed method on the recognition of negative emotion in each individual.

**FIGURE 8:**
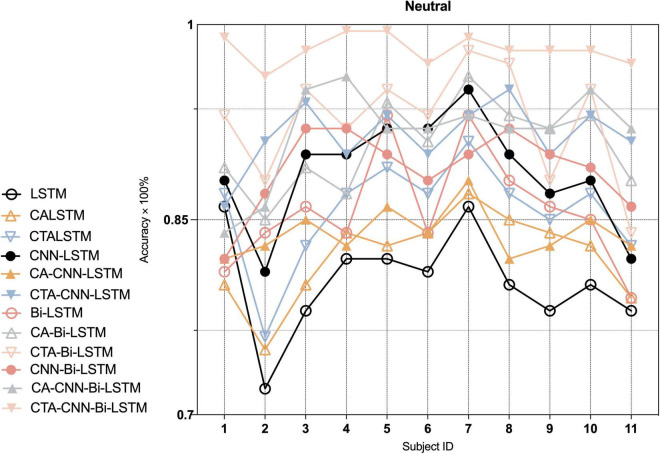
Average accuracy (%) of baseline and proposed method on the recognition of neutral emotion in each individual.

**FIGURE 9:**
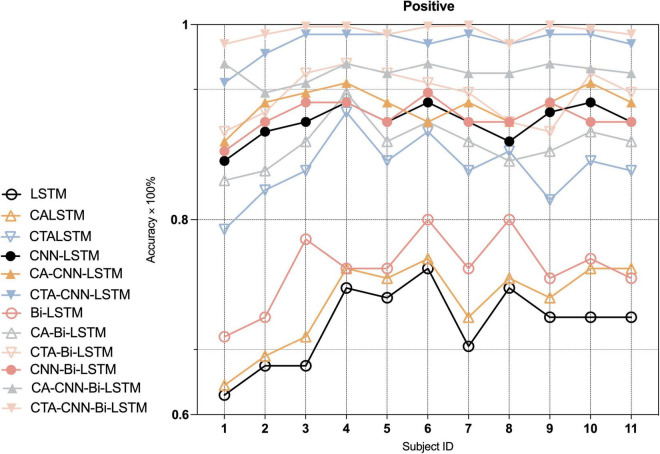
Average accuracy (%) of baseline and proposed method on the recognition of positive emotion in each individual.

Furthermore, to indicate the performance of the proposed framework on the emotion recognition of each individual, we conducted experiments on each individual. As Figures 7–9 show, the proposed framework CTA-CNN-Bi-LSTM almost achieved the best accuracy of emotion recognition for negative, neutral and positive emotions on each subject. In addition, for the base models, the RNN models did not perform well below 60% for each individual on negative emotions, but after adding the CTA module before the RNN models the individual’s negative emotion recognition rate with an accuracy of more than 80%. And the CNN-RNN series models perform better than the RNN series in terms of positive emotion for each subject. There was no such significant tendency in negative emotion and neutral emotion for each subject. For the negative emotion, the CA-Bi-LSTM model performed better than the CA-CNN-Bi-LSTM on subjects 5, 6, and 11. For the neutral emotion, the CTA-Bi-LSTM model performed better than the CTA-CNN-LSTM model on subjects 1, 5, 6, 7, and 8. However, the proposed framework performed best on each individual for the three emotions.

## Discussion

### Principal results and limitations

The experimental results reveal that the CTA-CNN-Bi-LSTM framework performs better in EEG emotion recognition as the proposed framework combined consideration of the spatial features and two directions’ temporal features which were extracted from the channels and temporal dimension of EEG signals most relevant to emotions.

In the first module of our proposed framework, the channel-temporal attention module applied to the clean EEG raw data emphasized meaningful feature information and suppresses irrelevant information in both channel and temporal dimensions. Firstly, the channel weights were calculated under the global average pooling and global maximum pooling in the temporal dimension to obtain two channel statistical descriptions (two different angles of the global field of view). In contrast, the channel attention module in the previous study (Tao et al., 2020) only conducted global average pooling on the temporal dimension (T8 and F8 dominated among 14 electrodes in the DREAMER dataset; FC5, P3, C4, and P8 dominated among 40 electrodes in DEAP), which may have resulted in the inability to distinguish the contribution of channels to different emotions. The channel weights in this study were calculated so that the weights of F3 and F4 achieved significant advantages in neutral emotion. For negative emotions, channel weights greater than 0.5 are F3, T7, F7, and T8, meanwhile the weights of channels F8 (0.2) and F7 (0.73) had a significant (*F* = 3.55, *p* < 0.01) in negative emotion, which both suggested that they played a major role in the channels corresponding to the left-brain area. For positive emotions, the channel weights greater than 0.5 were F8, T8, and P4, and channel P4 (0.6) and T7 (0.28) had a significant (*F* = 3.39, *p* < 0.01) in positive emotion, which indicate that the right-brain area corresponding to the channel was dominant. These findings are consistent with previous studies: (1) The valence theory stated that left-brain areas predominantly process negative emotions and right-brain areas process positive emotions (Demaree et al., 2005); (2) EEG signals in the frontal lobe, lateral temporal lobe, and parietal lobe brain regions of the brain were the most informative on different emotions (Lin et al., 2010; Zheng et al., 2017; Özerdem and Polat, 2017; Tong et al., 2018). If it is necessary to reduce electrodes while ensuring a high recognition rate of emotions, the intersection of all emotion-dominated channels or channels with significant differences can be selected. This means that F3, F4, F7, and F8 can be chosen for the task of our study. Other tasks can recalculate channel weights according to this method.

When the recoded EEG data obtained directly using the channel attention is used for subsequent model learning, as shown in Figure 5, the average accuracy of the CA-RNN/CA-CNN-RNN model improved only slightly compared to RNN/CNN-RNN, except the CA-CNN-LSTM model was slightly lower than the CNN-LSTM model. However, from Figure 6, the CA-CNN-LSTM improved the recognition accuracy of negative emotion by 10% over the CNN-LSTM model. The average accuracy of CTA-RNN/CTA-CNN-RNN not only increased but also achieved the minimum variance, demonstrating that the temporal attention mechanism did improve the representation of emotional state change time points in EEG signals while further suppressing the noise/artifact information. And the results were higher than the accuracy results of the previously mentioned related studies using channel selection (Alotaiby et al., 2015; Tong et al., 2018; Tao et al., 2020; Dura et al., 2021). Therefore, the EEG raw data was processed by the channel-temporal attention module to emphasize meaningful feature information and suppress irrelevant information in both channel and temporal dimensions.

In the second and third modules of our proposed framework, the recoded EEG signals (containing information on the most relevant channel and temporal dimensions to the task) from the channel-temporal attention module were fed into the CNNs and RNN to extract spatial and temporal features for emotion recognition. As Table 6 and Figure 5 shown, the training parameters of CNN-RNN without the channel-temporal attention mechanism (264,707/532,211) were much smaller than those of RNN (544,243/1108,963), while the average accuracy was substantially higher than that of RNN (19.34% and 21.29% improvement). This, as has been shown in previous studies (Sheykhivand et al., 2020; Zhang et al., 2020; Ramzan and Dawn, 2021), demonstrates that it is necessary to consider both spatial and temporal information of EEG signals for emotion recognition. And the CA-CNN-RNN models achieved an average accuracy of 78.11% (1.56% lower than CNN-LSTM model and 10% improvement on negative emotion) and 91.11% (3.37% improvement over CNN-Bi-LSTM model), respectively. It was further demonstrated that channel attention suppresses the information of irrelevant channels and enhances emotional information. Finally, the results of the CTA-CNN-Bi-LSTM model proposed in this study achieved the highest average accuracy of 98.75%. It further demonstrated that channel-temporal attention suppresses both the information of irrelevant channels and the irrelevant information of temporal dimensions. The CTA-CNN-Bi-LSTM model with an improvement of 7.25% over the CTA-CNN-LSTM model. The reason is that Bi-LSTM model learned the temporal information on the spatial feature map from both forward and reverse directions while LSTM model learned from only one direction in the forward direction. This is consistent with the conclusions in the study (Siami-Namini et al., 2019): Bi-LSTM model outperforms the LSTM model on temporal series forecasting tasks. As our experiments also employed 10-fold cross-validation, the average accuracy standard deviation values can more objectively demonstrate that the proposed framework has a high emotion recognition performance. From the result of confusion matrixes of negative emotion (Figure 6), it was found that the recognition rate of the basic RNN and CNN-RNN models on negative emotion was far lower than the other two emotions, firstly, the number of samples of negative emotion was lower than the other two emotions, and secondly, negative emotion seemed to be easily misclassified as neutral emotion. However, through the channel attention mechanism (CA) and the channel-temporal attention (CTA), the recognition of negative emotions with small samples is enhanced and the accuracy rate is further improved. Finally, we conducted experiments on each individual, and the proposed framework CTA-CNN-Bi-LSTM almost achieved the best accuracy of emotion recognition for negative, neutral, and positive emotions on each subject.

In summary, EEG raw 3s-dataset achieved the highest accuracy of 98.75% by the proposed method CTA-CNN-Bi-LSTM. It included channel-temporal attention module (CTA), spatial feature extraction (CNNs) and Bi-LSTM. The proposed method improved the average accuracy by 38.42% compared to the LSTM model. Of which, the channel-temporal attention module (CTA-CNN-Bi-LSTM) led to an average accuracy improvement of 11.01% for CNN-Bi-LSTM. The convolutional module (CNN-Bi-LSTM) resulted in an average accuracy improvement of 21.29% for Bi-LSTM. And the bi-directional LSTM module (CNN-Bi-LSTM) led to an improvement of 8.07% in CNN-LSTM. It indicates that the convolution module (spatial information of the EEG signal) provides the largest contribution (21.29%) to the accuracy improvement of the framework. The bi-directional LSTM module after the CNN module provides little enhancement (8.07%) to the framework. However, the addition of the channel-temporal attention module before the convolution module (by suppressing the irrelevant channel information and temporal dimensional noise) led to a further significant improvement (11.01%) in the accuracy of the model while reducing the std. dev. to a minimum. Thus, our proposed framework was demonstrated to be effective in extracting spatial and temporal information from recoded EEG signals (including most relevant channels and temporal dimensional information to emotion) for emotion recognition. However, our framework used the dataset divided using the subject-dependent method as the usage scenario of our task, and it has not been demonstrated whether the same high performance of emotion recognition can be achieved on the dataset divided by the subject-independent method.

### Conclusion and future work

The proposed framework in this paper used clean raw EEG signals (removal of muscle artifacts by MEMD-CCA) as input to an end-to-end deep learning method (without feature engineering) for emotion recognition. The proposed CTA-CNN-Bi-LSTM framework considered both spatial features and bidirectional temporal features in the channel dimension and temporal dimensions that were most relevant to emotions in the raw EEG signals. At first, the channel-temporal attention module suppresses the channel information in both the EEG signal that is not related to emotion and the noise in the spatial dimension in each channel. Later, the CNN-RNN module first extracts the spatial features in the recoded EEG signals and then feeds them into the Bi-LSTM network in order. Therefore, the Bi-LSTM learned the temporal information simultaneously from two directions (forward LSTM for previous information and reverse LSTM for future information) on the spatial feature maps. Finally, the results of four group experiments have demonstrated that CTA-CNN-Bi-LSTM improved EEG emotion recognition compared to other methods and achieved the highest average accuracy of 98.75% for negative, positive, and neutral emotion recognition. Therefore, the proposed framework reduces the emotion-independent information and noise in the channel and temporal dimensions, CTA-CNN-Bi-LSTM significantly improved the accuracy of emotion recognition in the dataset compared with existing methods.

However, this work may not achieve high emotion recognition accuracy for new individuals and requires retraining the model/fine-tuning the model to achieve it, which is not conducive to later applications of real-time emotion monitoring. In future work, after collecting EEG signals from more individuals, perhaps self-supervised learning models such as a contrastive learning model which learns knowledge on its own from unlabeled data, could be used to potentially realize a plug-and-play real-time EEG emotion recognition system. It focuses on learning the common features between similar examples and distinguishing the differences between non-similar examples to construct an encoder. This encoder has the ability to encode similar data of the same category and make the encoding results of different categories of data as different as possible.

## Data availability statement

The raw data supporting the conclusions of this article will be made available by the authors, without undue reservation.

## Author contributions

LJ and NK designed the experiments. LJ carried out the experiments. LJ, PS, and FZ analyzed the results. LJ and DC prepared the manuscript. All authors contributed to the article and approved the submitted version.
